# The prevalence, pattern and clinical presentation of developmental dental hard-tissue anomalies in children with primary and mix dentition from Ile-Ife, Nigeria

**DOI:** 10.1186/1472-6831-14-125

**Published:** 2014-10-16

**Authors:** Dada Oluwaseyi Temilola, Morenike Oluwatoyin Folayan, Olawunmi Fatusi, Nneka Maureen Chukwumah, Nneka Onyejaka, Elizabeth Oziegbe, Titus Oyedele, Kikelomo Adebanke Kolawole, Hakeem Agbaje

**Affiliations:** Obafemi Awolowo University Teaching Hospitals Complex, Ile-Ife, Nigeria; Obafemi Awolowo University, Ile-Ife, Nigeria

**Keywords:** Prevalence, Nigeria, Sex, Socioeconomic, Dental anomalies

## Abstract

**Background:**

The study of dental anomalies is important because it generates information that is important for both the anthropological and clinical management of patients. The objective of this study is to determine the prevalence and pattern of presentation of dental hard-tissue developmental anomalies in the mix dentition of children residing in Ile-Ife, a suburban region of Nigeria.

**Methods:**

Information on age, sex and socioeconomic status was collected from 1,036 children aged four months to 12 years through a household survey. Clinical examination was conducted to assess the presence of dental anomalies. Associations between age, sex, socioeconomic status, prevalence, and pattern of presentation of the developmental hard-tissue dental anomalies were determined.

**Result:**

Two hundred and seventy six (26.6%) children had dental anomalies. Of these, 23.8% had one anomaly, 2.5% had two anomalies, and 0.3% had more than two anomalies. Of the children with anomalies, 49.3%were male, 50.7%were female, and 47.8%, 28.6% and 23.6% were children from low, middle and high socioeconomic classes, respectively. More anomalies were seen in permanent than primary dentition. Anomalies of tooth structure were most prevalent (16.1%); anomalies which affect tooth number were least prevalent (1.3%). Dens evaginatus, peg-shaped lateral, macrodontia, and talon cusp were more prevalent in the permanent dentition, and dens evaginatus peg-shaped lateral and macrodontia were more prevalent in the maxilla. There were significantly more macrodontia anomalies in males and in children of high socioeconomic status.

**Conclusion:**

This large survey of dental hard-tissue anomalies found in the primary dentition and mixed dentition of children in Nigeria provides anthropological and clinical data that may aid the detection and management of dental problems of children in Nigeria.

## Background

Developmental dental anomalies are an important category of dental morphologic variations. Anomalies of shape, form, number and structure of the teeth may occur due to abnormal events in embryologic development. These events may be caused by genetic and environmental factors during the morpho-differentiation or histo-differentiation stages of tooth development [1]. Although asymptomatic, these anomalies can lead to clinical problems, including delayed or non-eruption of the normal series of teeth; attrition; breast feeding problems; compromised esthetics; occlusal interference; accidental cusp fracture; interference with tongue space, causing difficulty in speech and mastication; temporomandibular joint pain and dysfunction; malocclusion; periodontal problems because of excessive occlusal force; post-eruptive tooth breakdown; and increased susceptibility to caries [2–6].

Several studies [1, 7–10] have reported prevalence values for selected dental anomalies, including microdontia, talon cusps, congenitally missing teeth, supernumerary teeth, peg-shaped lateral incisors, fusion, gemination, and non-carious defects of enamel. Brook [10] reported a prevalence of 0.5% for microdontia, 1.6% for gemination and 0.1% for dens invaginatus in the primary dentition of children in Slough, England. Sex differences in the prevalence of these dental anomalies were not observed.

Hyperdontia in the primary dentition is rare [11]. However, there is significant racial difference in its incidence, ranging from 0.2% to 1.8% for Caucasians, compared with 7.8% for Mongoloids [12, 13]. Similar population differences have been reported also for hypodontia: a frequency of 0.4% was observed for Swedish children, which is midway of the range of 0.0% to 0.9% reported for Caucasians [12].

Little information is available about dental anomalies in any West African population. A few studies conducted in Nigeria focused only on very few developmental defects. Oredugba and Odukoya [14] reported a prevalence of 7.5% for chronological enamel hypoplasia. Before that, Adeniji [15] observed that the most prevalent dental anomaly observed clinically in school children in Lagos, Nigeria was enamel defect (10.4%), of which chronological enamel hypoplasia accounted for 6.7%. The prevalence of hypodontia in the permanent dentition was 0.4%, while that in primary dentition was 0.05% [15].

Data on dental anomalies are important for both the anthropological and clinical management of patients. The incidence and degree of expression of the anomalies can provide important information for phylogenic and genetic studies and help in the understanding of differences within and between populations [7]. Complications associated with dental hard- tissue anomalies include increased predisposition to caries and periodontal diseases, aesthetic impairment, pulpitis-induced pain, and crowding [2–6] all of which can negatively affect the oral health-related quality of life of affected children [15–17]. In view of this, it is important to conduct studies that could provide data on population-specific prevalence of dental anomalies. This is of specific importance in Nigeria, where evidence shows that the hard dental tissue profile of Nigerians differs from that of Caucasians [18, 19]. This study is an effort in that direction. The study determined the prevalence, pattern and clinical presentation of developmental anomalies in hard dental tissues in the primary dentition and mix dentition of children resident in Ile-Ife, a suburban region of Nigeria. It also examined the association between the presence of developmental anomalies in hard dental tissues, sex and socioeconomic status of the children.

## Methods

This cross-sectional study was conducted in Ile-Ife Central Local Government Area (LGA). According to the 2006 National Population Census, the population of the LGA was 138,818, with about 14,000 (10%) being children [20]. Recruitment of study participants was done at the National Population Enumeration sites in the LGA which had been for past national surveys [21, 22]. The enumeration sites in the LGA were used as recruitment sites because it was assumed that participants in these sites were familiar with the conduct of such surveys and, thus, were more likely to be open to discussions with the field workers.

### Study population

The study population included all children who were between four months and 12 years of age. Children excluded from the study were those who had a medical condition or syndrome associated with tooth anomalies, those who had cleft palate, and those with a history of diseases that could increase the risk for developing dental anomalies, such as maternal syphilis.

### Sample size

Sample size was estimated by use of the Leslie Fischer’s formula [23] for study populations of more than 10,000 at a 95% confidence level with a 50.0% prevalence [8] and a degree of freedom set at 0.05; sample size was 351. Based on a prevalence of 35.3% [8], it was determined that it would be necessary to examine a minimum of 993 children to identify 351 children with dental anomalies.

### Sampling technique

The sampling procedure was a multi-stage (three-level) cluster sampling aimed at selecting eligible persons: Stage 1, selection of enumeration areas within the LGA; Stage 2, enlistment of eligible individuals within households; Stage 3, selection of respondents for interview and examination. Enumeration areas in the LGA were also randomly selected. At the enumeration sites, every third house on each street was considered for recruitment of study participants. In each house, when more than one child was eligible for study, only one was selected. Eligibility was determined according to sex and age; male and female subjects were selected from consecutive houses, and the child who fell within the next age group was selected from each consecutive household. Recruitment of study participants continued in the enumeration site until the study sample was reached.

### Data collection tool

Data were collected by a personal interview method, using a structured questionnaire. A dentist conversant with normal and pathological dental features and who had been engaged in a similar household dental survey in the same LGA, was engaged as a field worker for the study.

Data collected included information on the child’s socio-demographic characteristics (age, sex, and socioeconomic status). Socioeconomic status for the purpose of this study was obtained through a multiple item scoring index [24] used in prior studies in Nigeria [25, 26]. The status designation combines the mother’s level of education with the occupation of the father; each child was allocated to a social class I to V, with social class V being lowest. Each child’s social class was classified as Class I (upper class), class II (upper middle class), class III (middle class), class IV (lower middle class) and class V (lower class).

All children eligible to participate in the study had an oral examination. The examinations were conducted under natural light, with the children sitting on a chair. The teeth were examined wet after debris had been removed by use of a piece of gauze.

### Ethical consideration

Ethical approval was obtained from the Obafemi Awolowo University Teaching Hospital Complex Ile-Ife. Approval for community entry was obtained from the LGA office. Written informed consent was obtained from a parent or legal guardian of each study participant prior to enrollment and assent was also sought from children who were 12 years of age.

### Questionnaire administration

Information on the socio-demographic profile of the children was obtained from either of the consenting parent or legal guardian and, where feasible, was corroborated by the child.

### Clinical examination

The diagnosis of dental hard-tissue anomaly was based exclusively on clinical examination. Detailed examination was conducted with sterile dental mirrors and probes. Gross debris was removed with gauze before examination of each tooth. The teeth present were charted. All dental anomalies observed were recorded. A tooth was considered present when any part of it was visible. A tooth present was scored as 1, and tooth absent as 0. Radiographs were not used. The following diagnostic criteria were used for the most common dental anomalies:

- Peg-shaped lateral: Any upper lateral incisor with a reduction in its mesio-distal size in a gingivo-incisal direction.
- Mesiodens: a supernumerary tooth present in the pre-maxilla between the two central incisors [7].
- Talon cusp: A prominent accessory cusp-like structure projecting incisally from the cingulum area of an incisor [8].
- Microdontia: Teeth which are physically smaller than usual [9].
- Macrodontia: Teeth which are physically larger than usual [9].
- Gemination: Anomalies which arise from an attempt at division of a single tooth germ by an invagination, with resultant incomplete formation of two teeth and corresponding increase in the number of teeth in the dental arch [7].
- Fusion: the union of two normally separated tooth buds with the resultant formation of a joined tooth with confluence of dentine [27].
- Enamel hypoplasia: is defined as a deficiency of enamel formation and is seen clinically as pits, grooves or generalized [28].
- Dens evaginatus: an accessory cusp whose morphology makes it an abnormal tubercle [29].
- Dens invaginatus: an invagination of enamel in the crown of the tooth [30].
- Supernumerary: an additional tooth to the normal series [31].
- Supplemental: an additional tooth to the normal series resembling the tooth with which it is associated [32].
- Hypodontia: The absence of a tooth or teeth, exclusive of the third molars [33].
- Tooth transposition: The positional interchange of two adjacent teeth [34].
- Notch shaped incisor: A condition of the teeth in which the incisal edge is notched and narrower than the neck area at the gums associated with maternal syphilis infection [35].

### Standardization of examiner

An intra-examiner reliability test was done to calibrate the principal investigator on consistency of diagnosis for dental anomalies. The test was done by examining pictures of various dental anomalies. The scoring for each of the pictures identified correctly was recorded and repeated twice at an interval of one week. The intra-examiner reliability score for each of the 16 dental anomalies studies was high.

### Data analysis

The ages of the study participants were divided into three categories for data analysis: ≤4 years, 5–8 years, and 9–12 years. The socioeconomic status of the children was also re-categorized into three classes: social classes I and II, high socioeconomic status; social class III, middle socioeconomic status; and social class IV and V, low socioeconomic status. Descriptive and bivariate analyses were conducted to test the association between dependent variables (presence of dental hard-tissue anomalies) and the child’s socioeconomic status and sex. Where appropriate, the Pearson’s Chi-squared test or Fisher’s exact test was used to calculate the test of association. Statistical analysis was done with Intercooled STATA (release 12) for windows. Simple proportions were computed. Statistical significance was inferred at p <0.05.

## Results

One thousand and thirty-six children were recruited into the study. No child eligible to participate in the study met the exclusion criteria. Age, sex, and socioeconomic class of study participants recruited for the study are highlighted in Table 1. Two hundred and seventy six (26.6%) children had dental hard-tissue anomalies. The most prevalent anomaly was hypoplastic enamel (16.1%), followed by dens evaginatus (6.4%). An anomaly of tooth structure was significantly more frequent than an anomaly of tooth size (16.1%vs 3.4% - p < 0.001), tooth shape (16.1 vs 8.4% - p < 0.001), and tooth number (16.1 vs 1.3% - p < 0.001). See Table 2. There was no significant sex difference in the prevalence of the anomalies, except for macrodontia: significantly more males than female had macrodontia (p < 0.001). Also, there was no significant difference in the prevalence of dental hard-tissue anomaly based on socioeconomic status, except for macrodontia: more children from the high socioeconomic status had macrodontia (p = 0.003). See Table 2.

**Table 1:** **Age, sex and socioeconomic status of study participants**

	Male (%)	Female (%)	Total (%)
			N = 1,036
**Age**			
≤4 years	193(37.4%)	199(38.3%)	392(37.8%)
5-8 years	199(38.6%)	189(36.3%)	388(37.5%)
9-12 years	124(24.0%)	132(25.4%)	256(24.7%)
Total	516(100%)	520(100%)	1,036(100%)
**Socioeconomic Status**			
Low	223(21.5%)	227(21.9%)	450(43.4%)
Middle	171(16.5%)	185(17.9%)	356(34.4%)
High	122(11.8%)	108(10.4%)	230(22.2%)
Total	516(49.8%)	520(50.2%)	1,036(100%)

**Table 2:** **Prevalence of dental anomaly by sex and socioeconomic status**

Dental hard-tissue anomaly	Number of cases affecting Male	Number of cases affecting Female	Number of cases affecting low SES	Number of cases affecting middle SES	Number of cases affecting high SES	*Prevalence of lesion
						N = 1,036
	n = 516	n =520	n =451	n =357	n =228	
Enamel hypoplasia	76(14.7%)	91(17.5%)	77(17.1%)	47(13.2%)	43(18.9%)	167(16.1%)
Dens evaginatus	33(6.4%)	33(6.3%)	38(8.4%)	17(4.8%)	11(4.8%)	66(6.4%)
Macrodontia	19(3.7%)	2(0.4%)	6(1.3%)	4(1.1%)	11(4.8%)	21(2.0%)
Peg shape lateral	9(1.7%)	7(1.3%)	4(0.9%)	8(2.2%)	4(1.8%)	16(1.5%)
Microdontia	5(0.9%)	10(1.9%)	4(0.9%)	7(2.0%)	4(1.8%)	15(1.4%)
Supernumerary	2(0.4%)	2(0.4%)	2(0.4%)	2(0.6%)	0(0.0%)	4(0.4%)
Fusion/Gemination	2(0.4%)	2(0.2%)	1(0.2%)	1(0.3%)	2(0.4%)	4(0.4%)
Supplemental	2(0.4%)	1(0.2%)	2(0.4%)	1(0.3%)	0(0.0%)	3(0.3%)
Talon cusp	1(0.2%)	2(0.4%)	2(0.2%)	0(0.0%)	1(0.3%)	3(0.3%)
Mesioden	1(0.2%)	0(0.0%)	0(0.0%)	0(0.0%)	1(0.3%)	1(0.1%)
Dens Invaginatus	1(0.2%)	0(0.0%)	1(0.2%)	0(0.0%)	0(0.0%)	1(0.1%)
Transposition	0(0.0%)	1(0.2%)	1(0.2%)	0(0.0%)	0(0.0%)	1(0.1%)
Notch incisor	0(0.0%)	1(0.2%)	1(0.2%)	0(0.0%)	0(0.0%)	1(0.1%)
Hypodontia	0(0.0%)	0(0.0%)	0(0.0%)	0(0.0%)	0(0.0%)	0(0.0%
Total	151(29.3%)	152(29.2%)	139(30.8%)	87(24.4%)	77(33.8%)	303(29.2%)

*number of persons with lesion divided by the number of study participants.

Significantly more cases of dental hard-tissue anomalies were identified in the permanent than in the primary dentition (5.4%vs 2.8%; p < 0.001). There were significantly more cases of densevaginatus (p < 0.001), macrodontia (p < 0.001), peg-shaped laterals (p < 0.001), talon cusp (p = 0.009) and notch shaped incisor (p = 0.002) in the permanent dentition than in the primary dentition. However, there were significantly more cases of hypoplastic enamel (p < 0.001) in the primary than permanent. See Table 3.

**Table 3:** **Prevalence of dental anomaly by type of dentition**

Dental hard-tissue anomaly	Number of primary tooth affected by lesion	Number of permanent tooth affected by lesion	*Prevalence of lesion
			N = 23,591
	n = 16,456	n = 7,135	
Hypoplastic enamel	371(2.3%)	254(3.6%)	625(2.7%)
Dens evaginatus	57(0.3%)	60(0.8%)	117(0.5%)
Macrodontia	0(0.0%)	34(0.5%)	104(0.4%)
Peg-shape lateral	8(0.05%)	16(0.2%)	24(0.1%)
Microdontia	13(0.08%)	9(0.1%)	22(0.09%)
Supernumerary	2(0.01%)	2(0.03%)	4(0.02%)
Fusion/Gemination	4(0.02%)	0(0.0%)	4(0.02%)
Supplemental	2(0.01%)	1(0.01%)	3(0.01%)
Talon cusp	0(0.0%)	3(0.04%)	3(0.01%)
Mesioden	0(0.0%)	1(0.01%)	1(0.004%)
Dens invaginatus	1(0.006%)	0(0.0%)	1(0.004%)
Transposition	0(0.0%)	2(0.03%)	2(0.008%)
Notch-shape incisor	0(0.0%)	4(0.06%)	4(0.02%)
Hypodontia	0(0.0%)	0(0.0%)	0(0.0%)
Total	458(2.8%)	386(5.4%)	844(3.6%)

*number of persons with lesion divided by the number of study participants.

Table 4 shows the number of teeth with anomalies in the maxilla and in the mandible. There were more lesions in the maxilla than in the mandible (4.4% vs 2.7%; p < 0.001). More children had dens evaginatus (p < 0.001), macrodontia (p = 0.002), peg-shaped laterals (p < 0.001) and notch shaped incisor (p = 0.04) in the maxilla than in the mandible. There were no significant differences in the number of teeth that had anomalies on the left side of the face when compared with the right (p = 0.77). See Table 5.

**Table 4:** **Number and percentage of teeth with dental anomalies in the maxilla and mandible**

Anomaly	Maxillary	Mandible	Total
	n = 11,732	n = 11,859	N = 23,591
Hypoplasia	324(2.8%)	301(2.5%)	625(2.7%)
Dens evaginatus	112(0.9%)	5(0.04%)	117(0.5%)
Macrodontia	32(0.3%)	2(0.02%)	34(0.1%)
Peg shaped lateral	23(0.2%)	1(0.008%)	24(0.1%)
Microdontia	13(0.1%)	9(0.08%)	22(0.09%)
Notch shaped incisor	4(0.03%)	0(0.0%)	4(0.02%)
Fusion/Gemination	3(0.03%)	1(0.008%)	4(0.02%)
Talon cusp	3(0.03%)	0(0.0%)	3(0.01%)
Supernumerary	2(0.02%)	2(0.02%)	4(0.02%)
Supplemental	1(0.008%)	2(0.02%)	3(0.01%)
Mesioden	1(0.008%)	0(0.0%)	1(0.008%)
Transposition	0(0.0%)	2(0.02%)	2(0.004%)
Dens invaginatus	1(0.008%)	0(0.0%)	1(0.008%)
Hypodontia	0(0.0%)	0(0.0%)	0(0.0%)
Total	519(4.4%)	325(2.7%)	844(3.6%)

**Table 5:** **Number and percentage of teeth with dental anomalies in the right and left sides of the jaws**

Anomaly	Right side (%)	Left side (%)	Total
	n = 11,776	n = 11,815	n = 23,591
Hypolasia	308(2.6%)	317(2.7%)	625(2.7%)
Dens evaginatus	64(0.5%)	53(0.4%)	117(0.5%)
Macrodintia	16(0.1%)	18(0.2%)	34(0.1%)
Peg shaped lateral	12(0.1%)	12(0.1%)	24(0.1%)
Microdontia	11(0.1%)	11(0.09%)	22(0.09%)
Notch shaped incisor	2(0.02%)	2(0.02%)	4(0.02%)
Fusion/Gemination	2(0.02%)	2(0.02%)	4(0.02%)
Talon cusp	2(0.02%)	1(0.008%)	3(0.01%)
Supernumerary	3(0.03%)	1(0.008%)	4(0.02%)
Supplemental	2(0.02%)	1(0.008%)	3(0.01%)
Transposition	2(0.02%)	0(0.0%)	2(0.008%)
Dens invaginatus	1(0.008%)	0(0.0%)	1(0.004%)
Hypodontia	0(0.0%)	0(0.0%)	0(0.0%)
Total	425(3.6%)	418(3.5%)	843(3.6%)

Of the 1,036 children examined, 247(23.8%) had at least one dental anomaly, 26(2.5%) had two anomalies, and 3(0.3%) had more than two. There was no difference in the number of male and female participants who had one or more dental hard-tissue anomalies. Significantly fewer children from the middle socio economic strata had two or more dental anomalies. See Table 6.

**Table 6:** **Prevalence of children with single and multiple hard dental tissue anomalies**

	Number (%) of male study participants with dental anomaly	Number (%) female study participants with dental anomaly	Total	Number (%) of study participants with dental anomaly in the low SES	Number (%) of study participants with dental anomaly In the middle SES	Number (%) of study participants with dental anomaly of in the high SES	Total
			N = 1036				N = 1036
	n = 516	n = 520					
						n = 228	
				n = 451			
					n = 357		
One anomaly	122(23.6%)	125(24.0%)	247(23.8%)	116(25.7%)	76(21.3%)	55(24.1%)	247(23.8%)
Two anomalies	13(2.5%)	13(2.5%)	26(2.5%)	15(3.3%)	2(0.6%)	9(3.9%)	26(2.5%)
More than two anomalies	1(0.2%)	2(0.4%)	3(0.3%)	1(0.1%)	1(0.3%)	1(0.4%)	3(0.3%)
**Total**	136(26.4%)	140(26.9%)	276(26.6%)	132(29.3%)	79(22.1%)	65(28.5%)	276(26.6%)

## Discussion

This study makes a unique contribution to the growing literature on the epidemiology of dental hard-tissue anomalies. Studies such as ours are important because of evidence of regional and racial disparity in the occurrence of dental anomalies. Currently, there is paucity of information from Nigeria on this subject, as previous studies examined only a limited number of dental hard-tissue anomalies.

This study has a methodological strength: it conducted a household survey, thus increasing the chances of including both in- and out-of-school children of all age groups and socioeconomic class. School-based studies in Nigeria have limited access to children from all socioeconomic strata since about 20% of children of primary-school age and 60% of children of secondary-school age are out of school. Nigeria accounts for 47% of the world’s out-of-school population [36].

Our study, however, had limitations. First, the study did not conduct radiographic examinations to rule out dental anomalies that could be present within the jaw bone, such as supplemental teeth, mesiodens, supernumerary teeth, dens invaginatus and hypodontia. Second, the diagnosis of microdontia and macrodontia was based on visual examination and not by measuring the dimensions of the teeth using casts; the dependence on visual examination for the diagnosis of these anomalies may have introduced bias. However, within the limits of the design of the study, the data still provide useful information that addresses the objective of the study.

We found that the prevalence of anomalies associated with number, form and size of dental hard tissues was low. Also, the prevalence of fusion/gemination was lower than the 1.9% previously reported in Nigeria [37], while the prevalence of macrodontia was higher than reported [38–40]. Significant sex and socioeconomic differences were also observed in the prevalence of macrodontia: the prevalence was higher in males and in those from the high socioeconomic strata. Brooks and Johns [41] had noted that males had a higher frequency of macrodontia in modern populations. The authors postulated that microdontia was an anomaly resulting from the interaction between genetic and environmental factors [42]. The association found between sex, socioeconomic status and macrodontia may further substantiate this postulation.

We reported one case of notched incisor. The mother of the child denied a history of syphilis. The child did not have other features of perinatal syphilis infection such as saddle nose, saber shins, protruding mandible, swollen knees. We decided to include this case in the study report since we could not exclude maternal syphilis as the etiological factor: there may therefore be other possible causes of notched incisors. However, we do realize that many families in Nigeria give birth in unorthodox health care centres; thus the diagnosis of maternal syphilis may have been missed. We may also have received a false-negative response to the question on syphilis.

Some of the study findings differ from those of prior reports. First, although this study, like a study in Nigeria [43], did not find a sex predilection for the prevalence of peg-shaped laterals, others [44–46] have reported a predilection for the condition in either male or female participants. Second, this study also reports a higher prevalence of dens evaginatus than had been reported in Mongoloids [47–49], although it found a similar prevalence to that observed in Hong Kong Chinese [50]. Prior studies had reported traits of dens evaginatus in blacks [51]. We were, however, not able to identify any study that reported on the prevalence of dens evaginatus in a predominantly black population. Third, the high prevalence of anomalies associated with dental structures reported in this study is not unusual, as prior studies had shown a greater prevalence of enamel hypoplasia in children from developing countries [52, 53] and in children with chronic or acute malnutrition [52, 53] or very low birth weight [54], which are common disorders in children from resource-limited settings. Studies conducted in metropolitan Nigerians reported a prevalence of enamel hypoplasia of 4% and 0.13% in primary dentition [55] permanent dentition [56, 57], respectively. The higher prevalence of enamel hypoplasia that we found in this study may reflect the more frequent exposure of children in this suburban setting to the various aetiological factors for enamel hypoplasia. Finally, our results suggest that the clinical presence of hypodontia in the mixed dentition is rare in this study population when the third molar is excluded from the surveyed dentition. This finding is contrary to the findings of Magnusson [58], and Amini, et al. [59], who found a high prevalence of hypodontia in the permanent dentition. Prior studies had highlighted the lower prevalence of hypodontia in the primary dentition when compared to the permanent dentition [60]. There, however, seemed to be no difference in the clinical presentation of hypodontia in the primary and mixed dentition of our study population.

## Conclusion

This large survey of dental hard-tissue anomalies in Nigerian children has provided anthropological and clinical data that may aid in the detection and management of dental problems in this nation’s children and perhaps elsewhere in the world. This information will enable paedodontists and public health specialists to prioritize screening measures for early diagnosis of childhood dental anomalies. Further studies may help in understanding the impact of these dental anomalies on the oral-health quality of life of the children.

## References

[1] Proffit WR, Proffit WR (1997). The development of orthodontic problems. Contemporary Orthodontics.

[2] Balcioğlu HA, Keklikoğlu N, Kökten G (2011). Talon cusp: a morphological dental anomaly. Rom J Morphol Embryol.

[3] Morinaga K, Aida N, Asai T, Tezen C, Ide Y, Nakagawa K (2010). Dens evaginatus on occlusal surface of maxillary second molar: a case report. Bull Tokyo Dent Coll.

[4] de Lima MV, Bramante CM, Garcia RB, Moraes IG, Bernardineli N (2007). Endodontic treatment of dens in dente associated with a chronic periapical lesion using an apical plug of mineral trioxide aggregate. Quintessence Int.

[5] Grošelj M, Jan J (2013). Molar incisor hypomineralisation and dental caries among children in Slovenia. Eur J Paediatr Dent.

[6] Hou GL, Lin CC, Tsai CC (1995). Ectopic supernumerary teeth as a predisposing cause in localized periodontitis. Case report. Aust Dent J.

[7] Bailit HL (1975). Dental variation among populations. An anthropologic view. Dent Clin North Am.

[8] Altug–Atac AT, Erdem D (2007). Prevalence and distribution of dental anomalies in orthodontic patients. Am J Orthod Dentofacial Orthoped.

[9] Tulunoglu O, Cankala DU, Ozdemir RC (2007). Talon’s cusp: report of four unusual cases. J Indian Soc Pedod Prev.

[10] Brook AH (1974). Dental anomalies of number, form and size: their prevalence in British school children. J Inst Ass Dent Child.

[11] Lehi G, Kaur A (2002). Supernumerary teeth in primary dentition of two cases. J Indian Soc Pedod Prev Dent.

[12] Grahnén II, Granath LE (1961). Numerical variations in primary dentition and their correlation with the permanent dentition. Odont Revy.

[13] Huang WH, Tsai TP, Su HL (1992). Mesiodens in the primary dentition stage: a radiographic study. J Dent Child.

[14] Orenuga OO, Odukoya O (2010). An epidemiological study of developmental defects of enamel in a group of Nigerian school children. Pesq Bras Odontoped Clin Integr, João Pessoa.

[15] Adeniji OO (1993). An Epidemiological Survey of Dental Anomalies in Nigerian School Children.

[16] Abanto J, Tello G, Bonini GC, Oliveira LB, Murakami C, Bönecker M (2014). Impact of traumatic dental injuries and malocclusions on quality of life of preschool children: a population-based study. Int J Paediatr Dent.

[17] Chaves AM, Rosenblatt A, Oliveira OF (2007). Enamel defects and its relation to life course events in primary dentition of Brazilian children: a longitudinal study. Community Dent Health.

[18] Otuyemi OD, Noar JH (1996). A comparison of crown size dimensions of the permanent teeth in a Nigerian and a British population. Eur J Orthod.

[19] Oziegbe EO, Adekoya-Sofowora C, Esan TA, Owotade FJ (2008). Eruption chronology of primary teeth in Nigerian children. J Clin Pediatr Dent.

[20] National Bureau of Statistics (2006). 2006 Population Census.

[21] Federal Ministry of Health (2008). National HIV/AIDS and Reproductive Health Survey, 2007 (NARHS Plus).

[22] Federal Ministry of Health (2010). National HIV Sero-prevalence Sentinel Survey among Pregnant Women Attending Antenatal Clinics in Nigeria.

[23] Araoye MO (2003). Research Methodology with Statistics for Health and Social Science.

[24] Bernard B, Robert Merton K (1957). Indices of Social Classification. Social Stratification-a Comparative Analysis of Structure and Process.

[25] Olusanya O, Okpere O, Ezimokhai M (1985). The importance of social class in voluntary fertility control in developing country. West Afr J Med.

[26] Folayan MO, Idehen EE, Ufomata D (2003). The effect of sociodemographic factors on dental anxiety in children seen in a suburban Nigerian hospital. Int J Paediatr Dent.

[27] Garvey MT, Barry HJ, Blake M (1999). Supernumerary teeth – an overview of classification, diagnosis and management. J Can Dent Assoc.

[28] Slayton RL, Warren JJ, Kanellis MJ, Levy SM, Islam M (2001). Prevalence of enamel hypoplasia and isolated opacities in the primary dentition. Islam M Pediatr.

[29] Levitan ME, Himel VT (2005). Dens Evaginatus: Literature Review, Pathophysiology, and Comprehensive Treatment Regimen.

[30] Neville B, Damm D, Allen C, Bouquot J (2002). Oral and Maxillofacial Pathology.

[31] Reddy YP, Karpagavinayagam K, Subbarao CV (2008). Management of dens invaginatus diagnosed by spiral computed tomography. J Endod.

[32] Salama FS, Abdel-Megid FY (1994). Supernumerary teeth: three case reports. Saudi Dent J.

[33] Gupta SK, Saxena P, Jain S, Jain D (2011). Prevalence and distribution of selected developmental dental anomalies in an Indian population. J Oral Sci.

[34] Cho SY, Chu V, Ki Y (2012). A retrospective study on 69 cases of maxillary tooth transposition. J Oral Sci.

[35] *The American Heritage Medical Dictionary*. Boston, MA : Houghton Mifflin Company; 2007.

[36] *UNICEF Nigerian-The Children-Education*. http://www.unicef.org/nigeria/children1937.html

[37] Oneyeaso CO, Oneyeaso AO (2006). Occlusal/dental anomalies found in a random sample of Nigerian schoolchildren. Oral Health Prev Dent.

[38] Altug-Atac AT, Erdem D (2007). Prevalence and distribution of dental anomalies in orthodontic patients. Am J Orthod Dento facial Orthop.

[39] Brook AH (1974). Dental anomalies of number, form, and size: their prevalence in British school children. J Int Assoc Dent Child.

[40] Ooshima T, Ishida R, Mishima K, Sobue S (1996). The prevalence of development anomalies of teeth and their association with tooth size in the primary and permanent dentition of 1650 Japanese children. Int J Pediatr Dent.

[41] Brooks AH, Johns CC, Ralanski R (1995). Dental anomalies of the number and size in a Romano-British population. Proceedings of the 100th International Symposium on Dental Morphology.

[42] Chung CS, Niswander JD, Runck DW, Bilben SE, Kau MCW (1972). Genetic and epidemiologic studies of oral characteristics in Hawaii’s school children: dental anomalies. Am J Phys Anthropol.

[43] Ucheonye I, Akeredolu T (2010). Prevalence of peg shaped laterals in south western Nigeria: a comparison of field and clinic findings. Int J Dent Sci.

[44] Meskin LH, Gorlin RJ (1969). Agenesis and peg shaped permanent maxillary lateral incisors. J Dent Res.

[45] Al-Emran S (1990). Prevalence of hypodontia and developmental malformations of permanent teeth in Saudi Arabia school children. Br J Orthod.

[46] Alvesal L, Portin P (1969). The inheritance pattern of missing, peg shaped and strongly mesiodistally reduced upper lateral incisors. Acta Odontol Scand.

[47] Wu KL (1955). Survey on mid-occlusal tubercles in bicuspids. China Stomatol Mag.

[48] Curzon ME, Curzon JA, Poyton HG (1970). Evaginated odontomes in the Keewatin Eskimo. Br Dent J.

[49] Merrill RG (1964). Occlusal anomalous tubercles on premolars of Alaskan Eskimos and Indians. Oral Surg.

[50] Cho SY, Ki Y, Chu V, Chan J (2006). Concomitant developmental dental anomalies in Chinese children with dens evaginatus. In J Paediatr Dent.

[51] Pearlman J, Curzon M (1977). An evaginated odontoma in an American Negro: report of a case. J Am Dent Assoc.

[52] Kanchanakamol U, Tuongratanaphan S, Tuongratanaphan S, Lertpoonvilaikul W, Chittaisong C, Pattanaporn K, Navia JM, Davies GN (1996). Prevalence of developmental enamel defects and dental caries in rural pre-school Thai children. Community Dent Health.

[53] Lukacs JR (1991). Localized enamel hypoplasia of human deciduous canine teeth: prevalence and pattern of expression in rural Pakistan. Hum Biol.

[54] Seow WK (1997). Effects of preterm birth on oral growth and development. Aust Dent J.

[55] Adenubi JO (1980). Dental Health Status of 4/5 year old children in Lagos private school. Nigeria Dental Journal.

[56] Sawyer DR, Taiwo EO, Mosadomi A (1984). Oral anomalies in Nigerian children. Community Dent Oral Epidemiol.

[57] Salako NO, Adenubi JO (1984). Chronological enamel hypoplasia. Trop Dent J.

[58] Magnusson TE (1977). Prevalence of hypodontia and malformations of permanent teeth in Iceland. Community Dent Oral Epidemiol.

[59] Amini F, Rakhshan V, Babaei P (2012). Prevalence and pattern of hypodontia in the permanent dentition of 3374 Iranian orthodontic patients. Dent Res J (Isfahan).

[60] Salama FS, Abdel-Megid FY (1994). Hypondontia of primary permanent teeth in a sample of Saudi children. Egypt Dent J.

[CR61] The pre-publication history for this paper can be accessed here: http://www.biomedcentral.com/1472-6831/14/125/prepub

